# Acute Compartment Syndrome Following a Low-Energy Ankle Injury: Case Report and Review of the Literature

**DOI:** 10.7759/cureus.103660

**Published:** 2026-02-15

**Authors:** Joshua Fernicola, William Sparks, Stephen Fernicola

**Affiliations:** 1 Orthopaedic Surgery, Medical College of Georgia at Augusta University, Augusta, USA; 2 Orthopaedic Surgery, University of South Florida, Tampa, USA

**Keywords:** compartment syndrome, gastrocnemius rupture, non-contact, orthopedic surgery, tennis leg, trauma

## Abstract

A healthy 21-year-old male active-duty sailor experienced acute compartment syndrome (ACS) of the right lower extremity after sustaining an inversion ankle injury during a summer football practice. In the emergency room, he was diaphoretic, with diffuse tenderness over the lateral leg and ankle and mild paresthesia over the dorsum of the foot. He had palpable pedal pulses and no pain with passive great toe extension. Initial radiographs demonstrated no acute osseous abnormality. However, the emergency department team ordered a creatine kinase (CK) out of concern for rhabdomyolysis. It returned elevated at 5,100 U/L. The patient was admitted for intravenous fluids, analgesics, and CK monitoring. In the morning, the patient reported minimal pain in his leg and ankle and improved numbness in his foot. His CK had decreased slightly to 5,018 U/L. He was discharged with a clinic follow-up appointment for the following morning. In the clinic, he reported worsening pain and numbness and an inability to dorsiflex his foot. He was immediately sent to the emergency room for management. On arrival, he exhibited tenderness to palpation of his anterior compartment musculature, as well as paresthesias and pain with passive extension of the big toe. After pressure measurements were obtained with an intracompartmental pressure monitoring needle, he was diagnosed with compartment syndrome and underwent dual-incision, 4-compartment fasciotomy, which led to symptom resolution. ACS should be ruled out when clinical suspicion exists, even if the mechanism of injury does not support it. This patient’s development of ACS after a non-contact injury represents a rare yet significant cause of ACS that should be considered in future patients with similar injuries.

## Introduction

Acute compartment syndrome (ACS) of the leg is a rare but potentially limb-threatening condition characterized by elevated compartment pressure [1]. Most commonly, it occurs as a result of high-energy injuries. The incidence of lower leg ACS is low, with one study reporting an occurrence rate of approximately 1.9% following tibial diaphyseal fractures [2]. While bony injuries are commonly observed in ACS, they have also been diagnosed following isolated gastrocnemius tears [3,4]. ACS is predominantly a clinical diagnosis with patients initially describing extreme pain of the affected extremity. As the intracompartmental pressure increases, patients can develop firm extremities, neurologic deficits, absent pulses, and pain with passive motion [5,6]. Modern technology allows for the measurement of compartment pressure, with one group suggesting 30 mmHg as a threshold necessitating urgent fasciotomy [7].

In this report, we present the case of a 21-year-old male who developed ACS after sustaining an atraumatic low-level inversion ankle injury during football practice.

## Case presentation

A healthy 21-year-old male active-duty sailor sustained a right ankle inversion injury during flag football practice. He immediately experienced a popping sensation, leg pain, and paresthesia over the dorsal aspect of the foot. In the emergency room, the patient was diaphoretic and endorsed diffuse muscle pain in his lower leg and ankle. He had worsening parasthesia over the dorsum of his foot but denied pain with passive extension of his big toe. Active extension and flexion of the big toe were normal. X-rays were obtained, which demonstrated no acute fracture. The emergency room team ordered a creatine kinase (CK) to investigate potential rhabdomyolysis, which was elevated at 5,100 U/L. He was admitted for intravenous (IV) fluids and observation. The following morning, he was found to have minimal tenderness to palpation of the lateral leg and ankle and was able to actively and passively flex and extend both the ankle and great toe. The parasthesia over the dorsal aspect of his foot was present but improving. A repeat CK was obtained due to the initially elevated value and found to have decreased to 5,018 U/L. With his improving examination and CK levels, he was discharged by the medicine team, with follow-up scheduled in the clinic the next morning.

The patient was observed in the outpatient medicine clinic the next morning and described severe pain in the right lower leg with worsening parasthesia and a new inability to dorsiflex at the ankle. He was immediately sent to the emergency room, where a repeat CK was found to be 13,419 U/L. He was admitted, started on IV fluids and analgesics, and the orthopaedic team was consulted.

Upon initial evaluation by the orthopaedic team, the patient reported severe pain to the lateral and anterior aspect of his leg, with the greatest severity around the middle third of his leg. He also reported pain in the lateral ankle but no pain in his foot or posterior leg. The lateral compartment of his leg was firm and tender. The anterior compartment was tense and tender, and the posterior compartment was soft and non-tender. Sensation to light touch was absent over the dorsal foot and diminished in the first web space but intact over the lateral, medial, and plantar aspects of the foot. He was unable to actively dorsiflex or evert the ankle. He was also not able to actively extend the great toe. He had severe pain with passive flexion of the great toe and plantar flexion and inversion of the ankle. During the interview, the patient could not tolerate lying in bed. He instead sat on the side of the bed with his foot resting on the floor. His blood pressure was 149/86 mmHg. An intracompartmental pressure monitoring needle was used to obtain compartment pressures, which were as follows: lateral 170 mmHg, anterior 39 mmHg, superficial posterior 13 mmHg, and deep posterior 12 mmHg.

**Figure 1:**
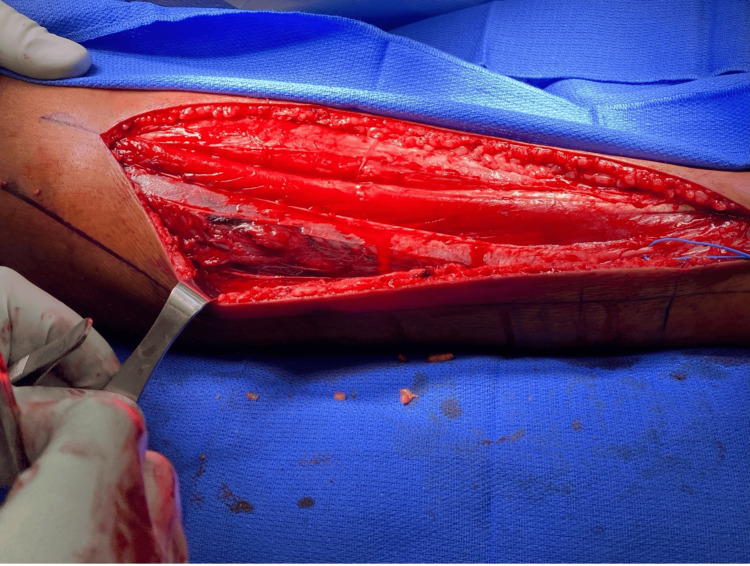
Intraoperative photograph obtained during the initial fasciotomy of the right lower extremity. An anterolateral incision provides exposure of the underlying musculature. The anterior compartment is directly visualized and appears pink, viable, and well perfused, consistent with preserved muscle viability following decompression. The lateral compartment is visible deep to the retracted tissue and appears comparatively darker and duskier than the anterior compartment. The superficial peroneal nerve is identified and protected with a blue vessel loop.

Given the elevated lateral and anterior compartment pressures, the patient was diagnosed with ACS and underwent emergent dual-incision, 4-compartment fasciotomy. The anterior compartment musculature was under significant pressure but was pink with normal consistency. It was also contractile to electrocautery stimulation. The lateral compartment was released in a similar fashion. The peroneus longus and brevis were under significant pressure and appeared gray and dusky, were relatively soft, and demonstrated no response to electrocautery stimulation. An image of the anterior and lateral compartments following initial fasciotomy is provided in Figure 1. There was also a moderate-sized hematoma and tear at the myotendinous junction of the peroneus longus. The hematoma was gently debrided, but the muscle was left to declare itself. The medial incision was made, and the superficial and deep posterior compartments were released. The muscles of the superficial and deep posterior compartments were pink, with normal consistency, and contractile to electrocautery stimulation. Both wounds were irrigated with normal saline. Wound vacs were placed over each incision, and he was placed in a splint.

**Figure 2:**
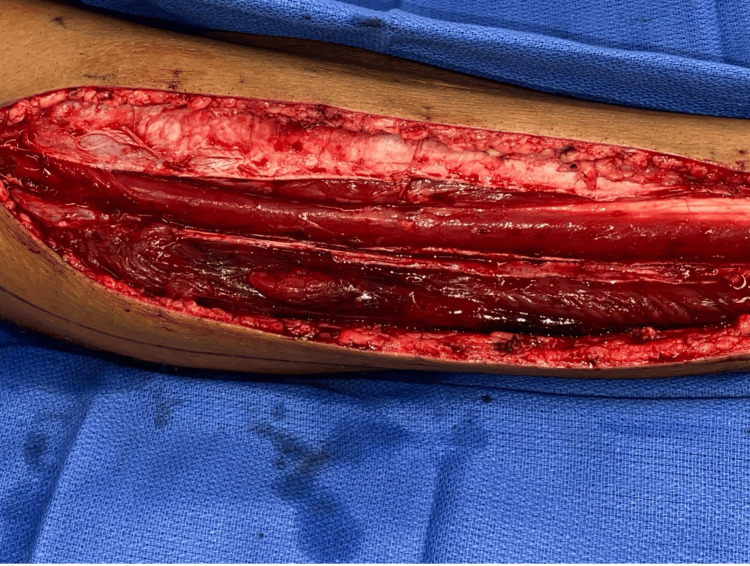
Intraoperative photograph obtained during the second operative intervention. Serial irrigation and debridement were performed through the original anterolateral incision. The musculature of the lateral compartment appears necrotic, and a substantial hematoma is evident along the length of the compartment.

**Figure 3:**
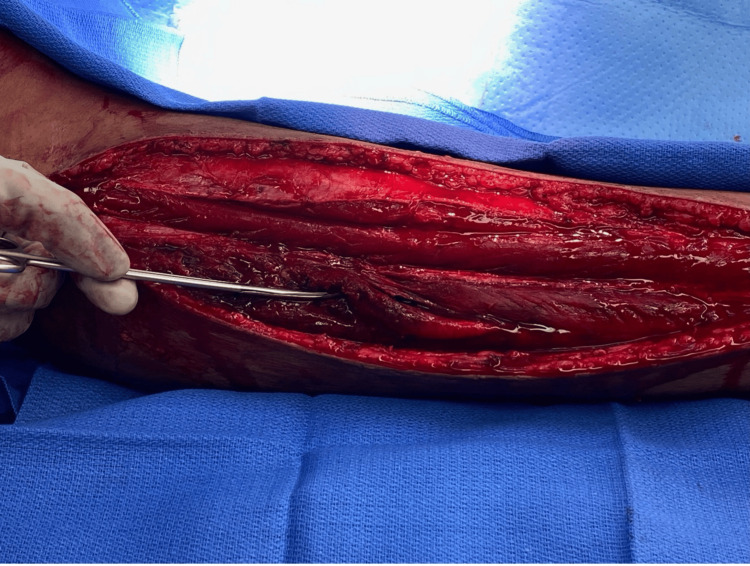
Intraoperative photograph obtained during debridement of the lateral compartment. The musculature appears non-viable, with weak and friable tissue that separated easily with gentle blunt dissection using scissors.

Following the initial fasciotomy, the patient underwent three serial irrigation and debridement procedures 2, 6, and 8 days later, respectively. These subsequent procedures were performed to evacuate hematoma, debride necrotic muscle, and assess the feasibility of skin-edge approximation for definitive closure. Images from the first irrigation and debridement are shown below (Figures 2, 3). The anterior compartment appeared healthy at every procedure. By the final procedure, over 90% of the peroneus longus and brevis tendons had been debrided. The peroneal tendons were then tacked down to aid as a static ankle stabilizer or to be available for tendon transfers later if needed. The superficial peroneal nerve was identified in its entirety in the leg and was found to be intact. Due to the large dead space, the lateral compartment was closed to prevent the formation of any future hematomas, and 1 g of vancomycin powder was placed within the compartment. Drains were placed both superficially and deeply. The skin was closed primarily, and an incisional vac was placed over both incisions.

Two weeks after final closure and suture removal, he was transitioned to a controlled ankle motion boot, allowed weightbearing as tolerated, and initiated physical therapy. At six weeks post-operation, the patient reported no pain and was able to actively dorsiflex the ankle and extend the big toe against resistance, and returned to normal shoes. At 5 months of follow-up, he had returned to full duty; however, his sensation in his foot did not improve.

## Discussion

ACS of the leg in adults is a limb-threatening emergency that demands rapid recognition and intervention. The diagnosis is fundamentally clinical, with the earliest and most sensitive sign being pain. This pain is typically severe, persistent, and often described as deep, aching, or burning, and is resistant to standard analgesic regimens. Escalating pain or pain refractory to medication should immediately raise suspicion for ACS. Pain on passive stretch of the muscles within the affected compartment is a highly specific clinical sign [8]. Paresthesia reflects nerve ischemia within the compartment and may precede motor deficits [9]. 

Motor deficits are considered late findings and indicate advanced ischemia with a high risk of irreversible tissue damage [9]. Physical examination may reveal a tense, firm, or swollen compartment, but subjective assessments of compartment tension may be unreliable, even among experienced clinicians [9]. Vascular signs such as pallor, pulselessness, and delayed capillary refill are late and unreliable indicators, as distal pulses are typically preserved until compartment pressures exceed systolic blood pressure [9].

Serial clinical examinations are essential, particularly for atypical patients like ours, as ACS is a dynamic process with symptoms evolving over time. The American College of Surgeons recommends sequential physical examinations every one to two hours for 24 to 48 hours in high-risk patients [9].

When clinical assessment is equivocal or unreliable, compartment pressure measurement becomes a critical adjunct. An intracompartmental pressure monitoring needle is the most widely used device for measuring compartment pressures, though there are multiple other options as well [8]. Historically, absolute compartment pressures between 30 and 45 mmHg were considered diagnostic for ACS and an indication for fasciotomy, but this approach has low specificity and risks unnecessary fasciotomies [10]. Instead, the recommended threshold is the differential pressure, defined as the difference between diastolic blood pressure and compartment pressure. A differential pressure of less than 30 mmHg is considered highly sensitive for ACS and is the recommended threshold for surgical intervention when adjunctive measurements are used to supplement an equivocal clinical examination [10]. 

A systematic review and meta-analysis by Lorange et al. found that the predictive value for diagnosing ACS was 21% for clinical signs alone and 29% for intracompartmental pressure monitoring alone. When both modalities were combined, the probability of accurate diagnosis increased to 68% [11]. This underscores the importance of a multimodal assessment. Intracompartmental pressure measurement should not be used in isolation but rather as an adjunct to clinical assessment, particularly in cases where the clinical examination is unreliable or equivocal.

Our literature review identified only a handful of reports describing ACS of the leg following low-energy or non-contact inversion ankle injuries in otherwise healthy individuals. Most commonly, it occurs as a result of high-energy injuries. The incidence of lower leg acute compartment syndrome is low, with one study reporting an occurrence rate of approximately 1.9% following tibial diaphyseal fractures [2]. Atraumatic presentations are markedly rarer, but some cases have been documented following gastrocnemius or soleus tears [4,5].

Even more uncommon are isolated lateral (peroneal) compartment syndromes after inversion injuries. Arciero and colleagues first reported anterolateral compartment syndrome secondary to peroneus longus rupture [12]. Rehman et al. described a case in which isolated lateral compartment syndrome developed late after a non-contact sports injury, with no muscle tear or hematoma [13]. Merriman et al. presented a 23-year-old collegiate football player who remained active despite a rupture of the peroneus longus muscle, ultimately developing compartment syndrome - a case very similar in mechanism to ours [14].

In our case, the mechanism aligned with those in the literature: Tearing of the peroneal musculature with hematoma formation led to increased swelling in the lateral compartment, resulting in dramatically elevated pressures (170 mmHg). These pressures far exceeded accepted thresholds and underscored the need for fasciotomy [7]. Notably, while the patient initially improved and was discharged after hospitalization, his subsequent return with worsening pain, paresthesia, and CK elevation (from 5,100 to 13,419 U/L) revealed the dynamic nature of ACS, underscoring the need to maintain a high index of suspicion even when early signs seem non-alarming.

The long-term prognosis for patients undergoing fasciotomies for ACS is dependent on the degree of local soft tissue damage. Foot drop and ankle contractures can often be sequelae with significant damage to the anterior and posterior compartments. In our patient with the loss of the peroneal muscles, the largest concern would be the lack of ankle eversion and loss of dynamic ankle stability. The goal of leaving the tendons is that they would function as a static stabilizer, preventing him from having any clinical ankle instability. We elected not to fully anchor them into the bone, so they could still be used for tendon transfers if needed. Despite a residual superficial peroneal sensory deficit, the patient achieved full ankle dorsiflexion and great toe extension by six weeks and returned to full duty by five months. As of this publication, he has not had any issues with ankle instability. 

This case adds to growing evidence that ACS can occur without fracture or significant trauma, resulting instead from inversion injuries leading to muscle tears and hematoma formation. Clinicians must remain vigilant for signs of ACS and consider compartment pressure measurement or repeat examination when clinical signs and symptoms are concerning despite a low-energy mechanism of injury.

## Conclusions

This patient’s atraumatic ACS developed after a low-energy inversion ankle injury. This uncommon mechanism likely increases the risk of delayed diagnosis. His presentation highlights the importance of maintaining a high index of suspicion for ACS, even in the absence of fracture or high-energy trauma. Early recognition and prompt surgical intervention were critical in preventing further functional loss and achieving a favorable recovery. Despite extensive peroneal muscle debridement and residual sensory deficits, the patient regained strength and returned to full duty, underscoring the effectiveness of timely fasciotomy combined with staged wound management. This case adds to the limited literature on non-contact, inversion injury-related ACS and may guide future clinicians when evaluating patients with similar mechanisms of injury.
